# High‐frequency oscillations mirror severity of human temporal lobe seizures

**DOI:** 10.1002/acn3.50941

**Published:** 2019-11-21

**Authors:** Jan Schönberger, Nadja Birk, Daniel Lachner‐Piza, Matthias Dümpelmann, Andreas Schulze‐Bonhage, Julia Jacobs

**Affiliations:** ^1^ Universitätsklinikum Freiburg Epilepsiezentrum Breisacher Straße 64 79106 Freiburg im Breisgau Germany; ^2^ Klinik für Neuropädiatrie und Muskelerkrankungen Universitätsklinikum Freiburg Mathildenstraße 1 79106 Freiburg im Breisgau Germany; ^3^ Berta‐Ottenstein‐Programme Faculty of Medicine University of Freiburg Freiburg Germany

## Abstract

**Objective:**

Many patients with epilepsy have both focal and bilateral tonic‐clonic seizures (BTCSs), but it is largely unclear why ictal activity spreads only sometimes. Previous work indicates that interictal high‐frequency oscillations (HFOs), traditionally subdivided into ripples (80–250 Hz) and fast ripples (250–500 Hz), are a promising biomarker of epileptogenicity. We aimed to investigate whether HFOs correlate with the emergence of seizure activity and whether they differ between focal seizures (FSs) with impaired awareness and BTCSs.

**Methods:**

We retrospectively analyzed 15 FSs and 13 BTCSs from seven patients with mesial temporal lobe epilepsy, each of them with at least one BTCS and at least one FS. Representative intervals of intracranial electroencephalography from the seizure onset zone (SOZ) and remote non‐SOZ areas were selected to compare pre‐ictal, complex focal, tonic‐clonic, and postictal periods. Ripples and fast ripples were visually identified and their density, that is, percentage of time occupied by the respective events, computed.

**Results:**

Ripple and fast ripple densities increased inside the SOZ after seizure onset (*P* < 0.01) and in remote areas after progression to BTCSs (*P* < 0.01). Postictal SOZ ripple density dropped below pre‐ictal levels (*P* < 0.001). Prior to onset of bilateral tonic‐clonic movements, ripple density inside the SOZ is higher in BTCSs than in FSs (*P* < 0.05).

**Interpretation:**

Ripples and fast ripples correlate with onset and spread of ictal activity. Abundant ripples inside the SOZ may reflect the activation of specific neuronal networks related to imminent spread of seizure activity.

## Introduction

Seizures are not only the key feature defining epilepsy,^1^ their occurrence is also a major determinant of patients' safety and quality of life. This applies in particular to seizures that culminate in bilateral tonic‐clonic movements.^2^ To improve treatment of individual patients, we aim for (1) understanding the mechanisms underlying seizure generation and spread,(2) identification of biomarkers that correlate with seizure severity and even more (3) have a prognostic value regarding the progression to a bilateral tonic‐clonic seizures (BTCS).

Previous work indicates that analysis of high‐frequency oscillations (HFOs) might help us tackle these problems – both from a basic science and from a clinical perspective. According to animal model studies, HFOs reflect key stages of pathophysiology in epileptogenic networks. The two traditionally distinguished subtypes have been linked to clearly discernible mechanisms: While ripples are likely due to synchronous firing coordinated by inhibitory currents^3^, ^4^, ^5^ (see^6^ for a review), fast ripples might mirror in‐ and out‐of‐phase firing of different pyramidal cell clusters^7^, ^8^, ^9^ (see^10^ for a review). Moreover, HFOs are promising biomarkers of epileptogenicity. Several studies in epilepsy surgery patients suggest that resection of HFO‐generating areas is associated with seizure‐free outcome,^11^, ^12^, ^13^, ^14^ thus indicating their potential for delineation of the epileptogenic zone. Besides, HFO rates increased after reduction of antiepileptic medication.^15^ It seems noteworthy, however, that these studies are based on interictal data. Evidence on ictal HFOs in humans is comparably limited, even though high‐frequency activity was initially described at the start of seizures.^16^ This may at least partly be due to the fact that sharp transients and wideband amplitude increases can make identification of “true” HFOs challenging.^17^ Only recently, the relationship between HFOs and electroencephalography (EEG) seizure onset patterns has been studied.^18^, ^19^

We aimed to address this gap of knowledge in patients with mesial temporal lobe epilepsy (MTLE), which is the most frequent focal epilepsy.^20^ Specifically, we asked whether ripple and fast ripple densities correlate with seizure severity and whether they might be useful biomarkers for predicting progression to BTCSs.

## Methods

### Patient selection

We considered all patients with drug‐resistant MTLE who, as part of their evaluation for epilepsy surgery, had undergone intracranial EEG recordings at the Freiburg Epilepsy Center between 2010 and 2016. From these, subjects with a video‐EEG recording of at least one focal seizure (FS) with impaired awareness and one BTCS were selected. This study was approved by the Ethics Commission at the University Medical Center Freiburg and written informed consent was obtained from all patients.

### Selection of seizures and intervals

Seizure onset was defined as the start of a mesial temporal lobe seizure onset pattern, consisting typically of repetitive high‐amplitude spikes or low‐voltage fast activity.^21^ BTCSs were required to be characterized by clear tonic‐clonic movements bilaterally. As has been suggested previously, we divided them into a focal part and a bilateral tonic‐clonic part (bilat. TC).^22^ If bilateral tonic limb posturing or behavioral vocalization occurred before bilateral tonic‐clonic movements, this was considered to be the onset of the bilateral tonic‐clonic part.^23^

**Figure 1:**
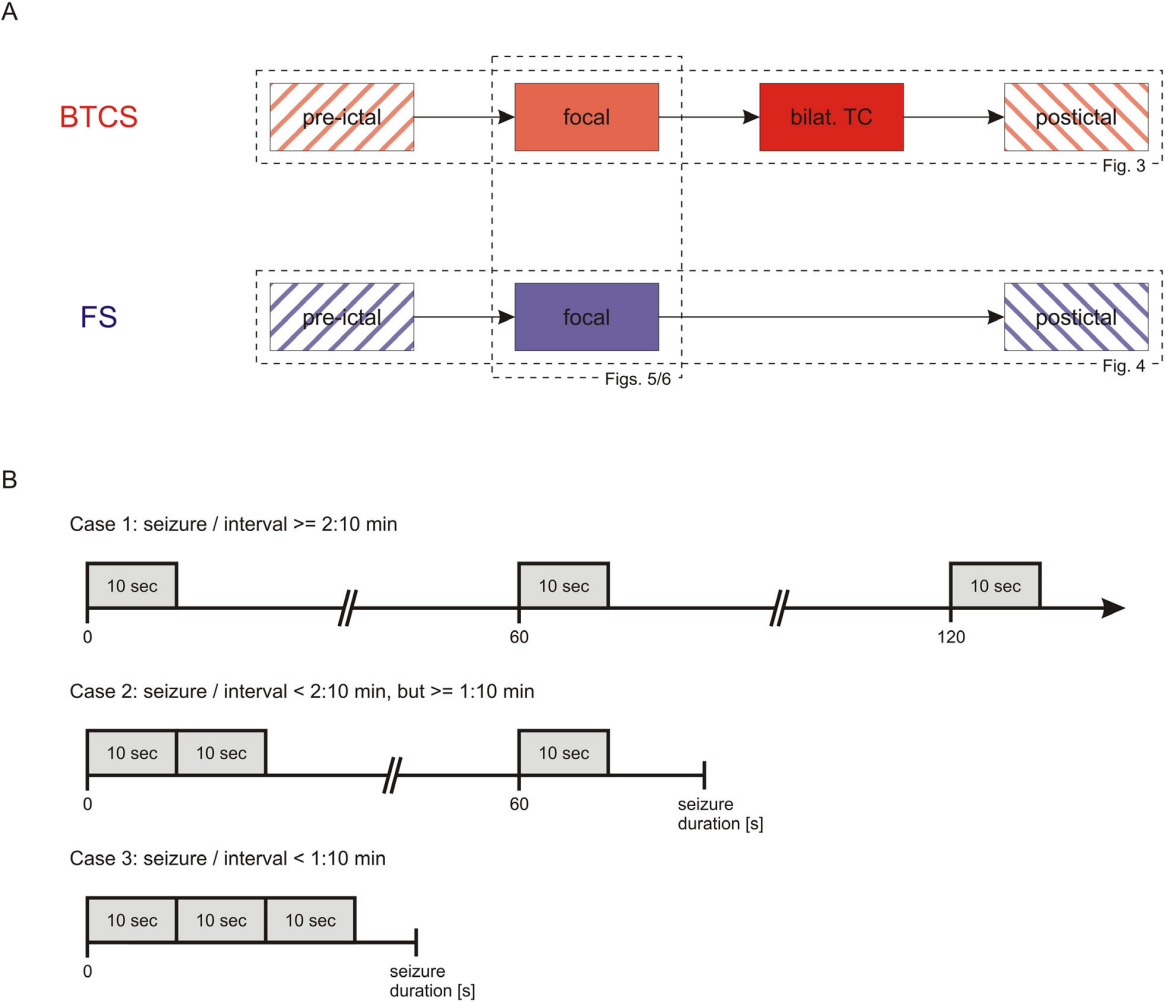
Study design. (A) Schematic illustration of our approach. We analyzed HFOs during the course of bilateral tonic‐clonic seizures (BTCS; Fig. 3) and focal seizures with impaired awareness (FS; Fig. 4) and compared analogous parts (Figs. 5 and 6). (B) Selection of 30 sec intervals from focal and bilateral tonic‐clonic parts. Three 10 sec segments were chosen to obtain data representative of the whole part irrespective of its duration. We distinguished three cases. See methods section for a detailed description.

We aimed to analyze data representative of the different stages of a seizure. 30 sec intervals were therefore selected as follows (Fig. 1):

(1) Pre‐ictal: 30 sec prior to seizure onset
(2a) Focal part (focal_FS_ and focal_BTCS_): If this stage was
  - longer than 2:10 min: Initial 10 sec of each of the first 3 min after seizure onset
  - shorter than 2:10 min, but longer than 1:10 min: Initial 20 sec of the first and initial 10 sec of the second minute
  - shorter than 1:10 min: Initial 30 sec after seizure onset
2b Bilateral tonic‐clonic part: same procedure as for (2a), but after onset of bilateral tonic‐clonic movements
3 Postictal: 30 sec after the end of ictal activity

We therefore only included FSs of at least 30 sec duration and BTCSs with focal and bilateral tonic‐clonic parts of at least 30 sec each. Seizures that arose less than 1 h after a previous seizure were excluded. A maximum of three seizures per patient of either type were included to minimize bias toward patients with many seizures.

### Intracranial EEG recordings and selection of channels

Intracranial electrodes (Ad‐Tech Medical Instrument Corporation, Racine, WI, USA) had been implanted based on their estimated value for clinical decision‐making in the individual patient. The mesial temporal lobe was investigated by at least two stereotactic depth electrodes (hippocampus and amygdala) in five patients and one depth electrode (hippocampus) plus a subdural grid and several strip electrodes in two patients. Intracranial EEG was recorded using Profusion EEG (Compumedics Limited, Abbotsford, Victoria, Australia). The sampling rate was 2 kHz and recordings were band‐pass filtered from 1.6 to 800 Hz.

Intracranial electrode contacts with a clearly ictal pattern within 2 sec of seizure onset were considered as representative of the seizure onset zone (SOZ). They were selected by a neurologist as part of the clinical routine and thus independently of this research study. We also selected five bipolar channels remote from the SOZ in each patient (“non‐SOZ”).

### HFO identification

HFOs were visually identified by two reviewers independently based on previously established procedures.^11^, ^15^, ^24^ In brief, we used the Harmonie Reviewer software to review bipolar montages of neighboring intracranial electrode contacts. The screen was vertically split, and finite impulse response‐filtered traces were displayed at maximum temporal resolution (0.4 sec on each half) to mark ripples (80–250 Hz) on the left and fast ripples (>250 Hz) on the right. An event was only regarded as an HFO if consisting of at least four oscillatory cycles. Two events were regarded as distinct if separated by at least two non‐HFO cycles,^11^ that is, without clear oscillatory activity for an interval corresponding to at least two cycles of the, respectively, analyzed oscillation. HFOs that co‐occurred with epileptic spikes were included, but we took great care to exclude events associated with artificial sharp transients.^17^

### Data analysis

Further computations were performed with custom‐written routines in Matlab (Mathworks, Natick, MA). HFO density was defined as the percentage of time occupied by HFOs, as described previously.^15^, ^18^ For group analyses, we considered the bipolar channel with maximum HFO density as representative of the, respectively, examined seizure, interval, and location (“SOZ” vs. “non‐SOZ”).

### Statistical hypothesis testing

The data were considered to be not normally distributed. We therefore specified the median as a measure of central tendency and the range as a measure of dispersion. The two‐sided Wilcoxon signed‐ranks test was applied for paired data and the two‐sided Wilcoxon rank sum test for unpaired data. The Holm‐Bonferroni method was used to correct for multiple comparisons. A significance level of 5% was chosen.

## Results

### Seizure and patient characteristics

**Figure 2:**
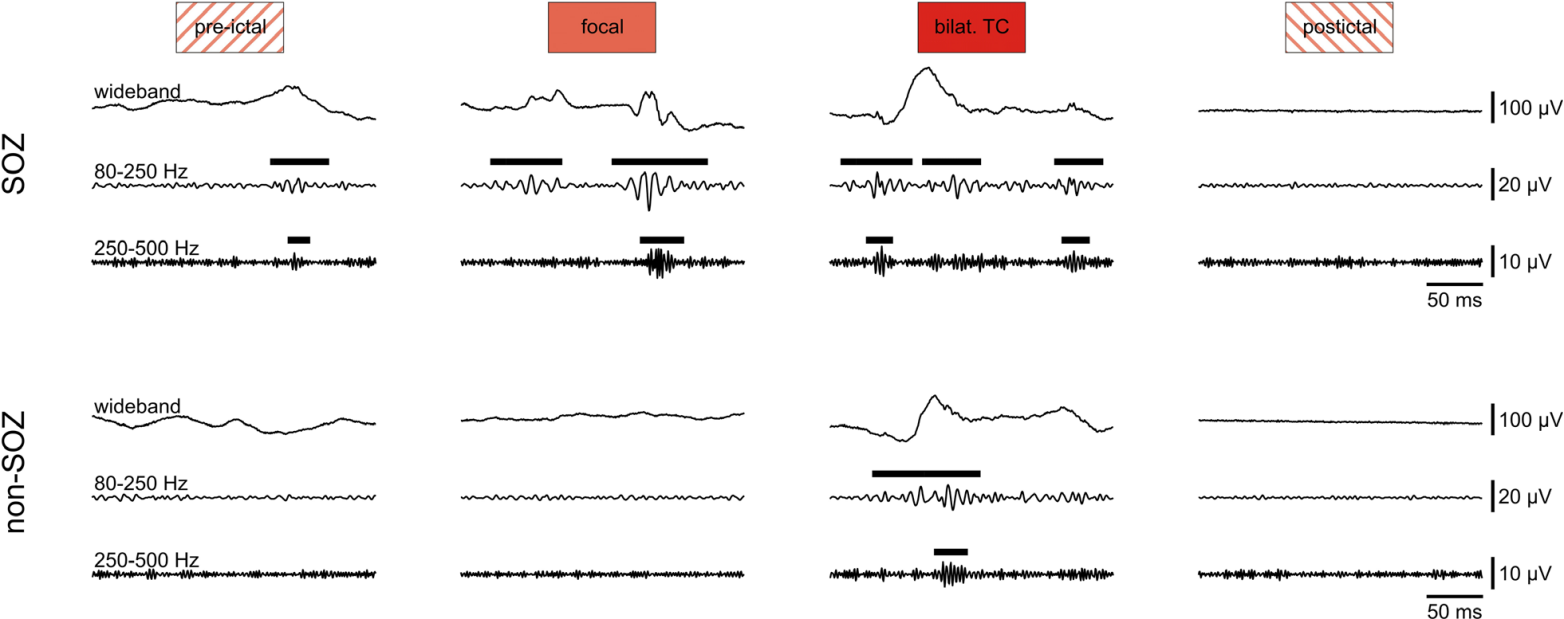
Representative examples of visually identified ripples and fast ripples during the course of a BTCS. Bold horizontal bars above band‐pass filtered traces indicate event duration. Data were selected from the same seizure and from the same SOZ and non‐SOZ channels.

We reviewed 119 consecutive patients with drug‐resistant focal epilepsy who, as part of their evaluation for epilepsy surgery, had undergone intracranial EEG recordings. Fifteen FSs and 13 BTCSs from seven patients (six females, one male; age: median 37 years, range 22–50 years; see Table 1 for more clinical data) fulfilled the inclusion criteria. Clinical and EEG findings were always suggestive of a mesial temporal SOZ. Representative examples of visually identified HFOs are shown in Figure 2.

**Table 1:** Clinical data.

ID	Sex	Age	MRI	SOZ	N_FS_	N_BTCS_	Semiology prior to progression to a BTCS
1	m	50	H head + A enlargement R	H ant, A R	4	1	Oral automatisms
2	f	39	H sclerosis R	H ant, H post, A R	3	6	Oral + manual automatisms, focal tonic, vocalization
3	f	37	s/p temporal pole resection without AHE R	H ant, A R	1	3	Oral automatisms, focal tonic
4	f	52	H sclerosis L	H L	5	1	Oral + manual automatisms, focal tonic
5	f	29	FCD MTL L	H ant, H post, A L	6	6	Oral + manual automatisms
6	f	23	normal	H L	1	2	Speech arrest, vocalization
7	f	22	H atrophy R	H ant + A R	2	1	Oral + manual automatisms, focal tonic, vocalization

A, amygdala; ant, anterior; FCD, focal cortical dysplasia; H, hippocampus; L, left; MTL, mesial temporal lobe; N_FS_/ N_BTCS_, number of FSs/BTCSs during evaluation period, post, posterior; R, right; s/p, status post.

### Ripples during the course of seizures

**Figure 3:**
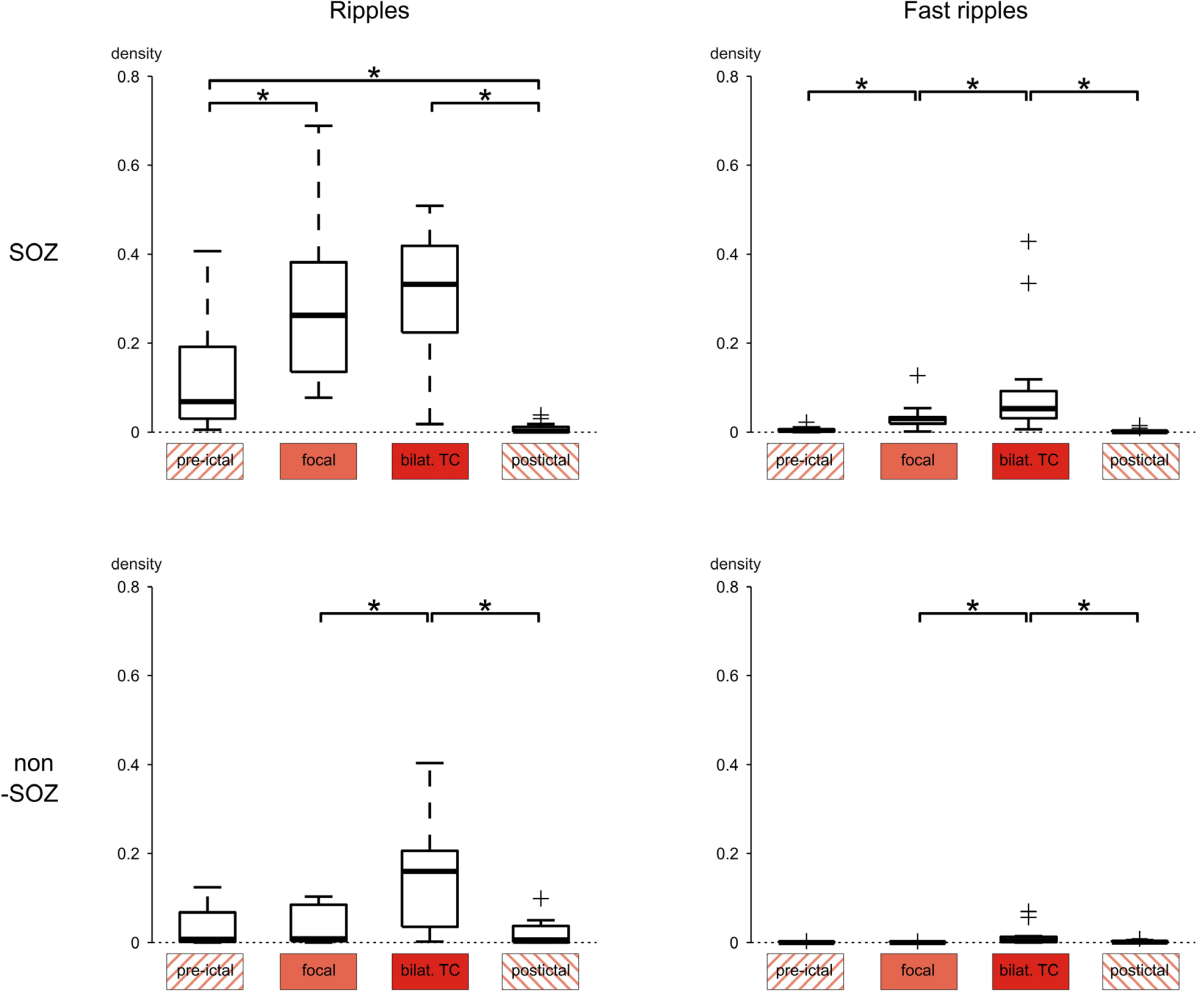
HFOs during the course of BTCSs. Ripple density increased after seizure onset inside the SOZ (left, top; *P* = 0.003) and after progression to a BTCS in remote areas (left, bottom; *P* = 0.008). Postictal intervals were characterized by a pronounced drop (SOZ: *P* < 0.001; non‐SOZ: *P* = 0.003). Note the suppression of SOZ ripples compared to pre‐ictal levels (*P* < 0.001). Fast ripple density inside the SOZ increased in parallel to the clinical progression of seizures (right, top; pre‐ictal vs. focal: *P* < 0.001; focal vs. bilat. TC: *P* = 0.04), and only after progression to a BTCS in remote areas (right, bottom; *P* = 0.001). Postictal intervals were characterized by a pronounced drop (SOZ: *P* < 0.001, non‐SOZ: *P* = 0.03), but not below pre‐ictal levels. *indicates a statistically significant difference.

**Figure 4:**
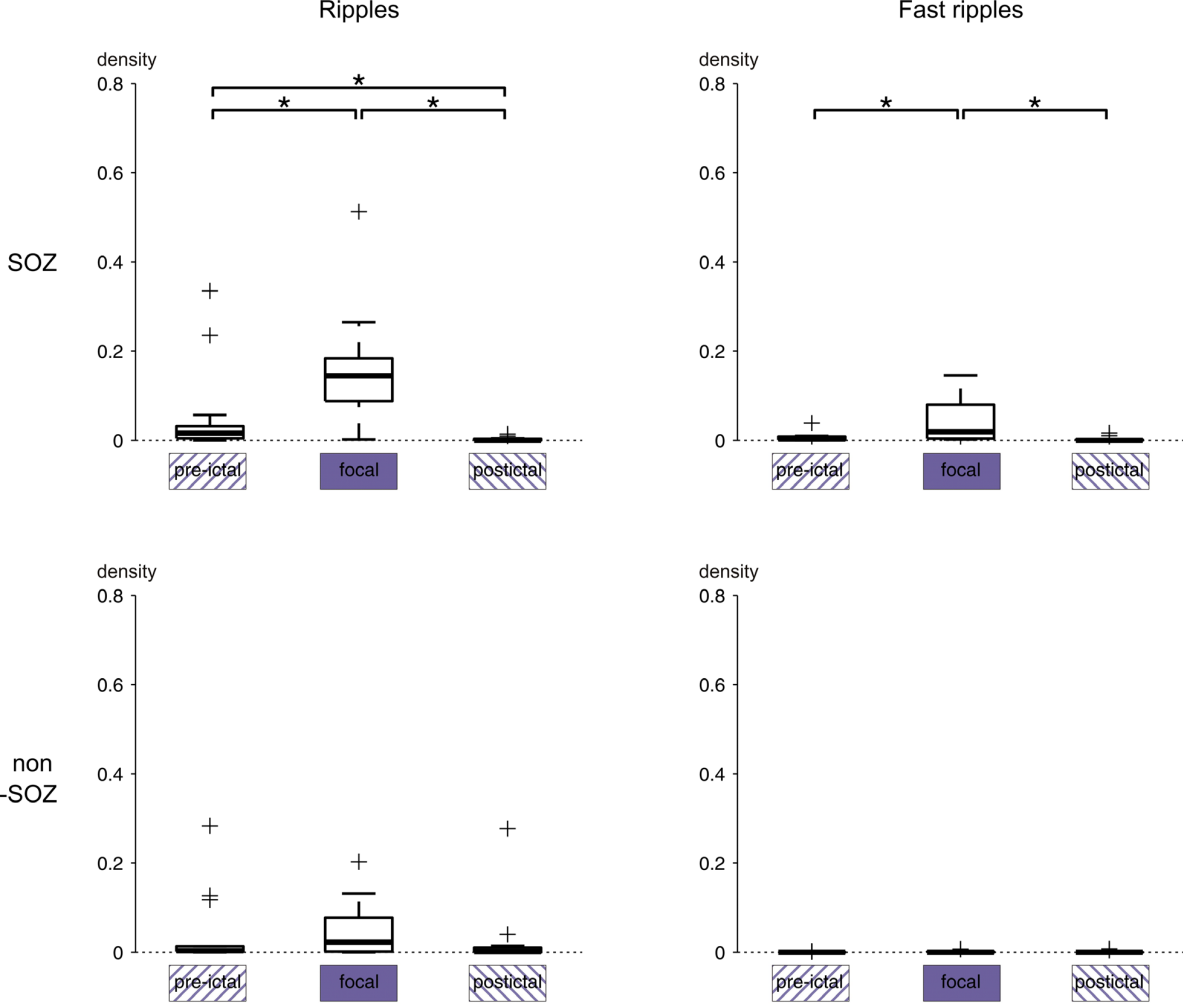
HFOs during the course of FS. Ripple density (left, top; *P* = 0.002) and fast ripple density (right, top; *P* < 0.001) inside the SOZ (top) increased after seizure onset. Postictal intervals were characterized by a drop (ripples: *P* < 0.001; fast ripples: *P* < 0.001). Note the suppression of SOZ ripples compared to pre‐ictal levels (*P* < 0.001). No significant differences were found in remote areas (bottom). *indicates a statistically significant difference.

HFO densities were first compared between different stages of BTCSs (Fig. 3; *P* values specified in the text below) and FSs (Fig. 4; *P* values specified below if inconsistent with findings from BTCSs). Seizure onset was associated with a significant increase in ripple density inside the SOZ (*P* = 0.003, Wilcoxon matched‐pairs signed‐ranks test), but not in selected remote areas (*P* = 0.62). After progression to the bilateral tonic‐clonic part, in turn, ripple density increased only in these non‐SOZ regions (*P* = 0.008), while remaining stable inside the SOZ (*P* = 1). Postictal intervals were characterized by a marked decrease in ripple density inside the SOZ (*P* < 0.001) and in BTCSs also remote from it (*P* = 0.003; FS: *P* = 0.06). In either subgroup, ripple density dropped below pre‐ictal levels inside the SOZ (*P* < 0.001), but not in non‐SOZ areas (*P* = 0.52).

### Fast ripples during the course of seizures

In analogy with ripple density, seizure onset was followed by a distinct increase in fast ripple density inside the SOZ (*P* < 0.001, Wilcoxon matched‐pairs signed‐ranks test), but not in non‐SOZ regions (*P* = 0.81) – irrespective of whether or not seizures generalized (Figs. 3 and 4). After progression to a BTCS, fast ripple density increased globally, that is, both inside (*P* = 0.04) and remote from the SOZ (*P* = 0.001). Postictal intervals were characterized by a pronounced decrease in fast ripples (SOZ: *P* < 0.001, non‐SOZ: *P* = 0.03), which was specific to the SOZ in FSs (SOZ: *P* < 0.001, non‐SOZ: *P* = 0.84). In none of the subgroups, however, fast ripple density dropped below pre‐ictal levels (SOZ: *P* = 0.07, non‐SOZ: *P* = 0.23).

### Comparison of FS versus BTCS

**Figure 5:**
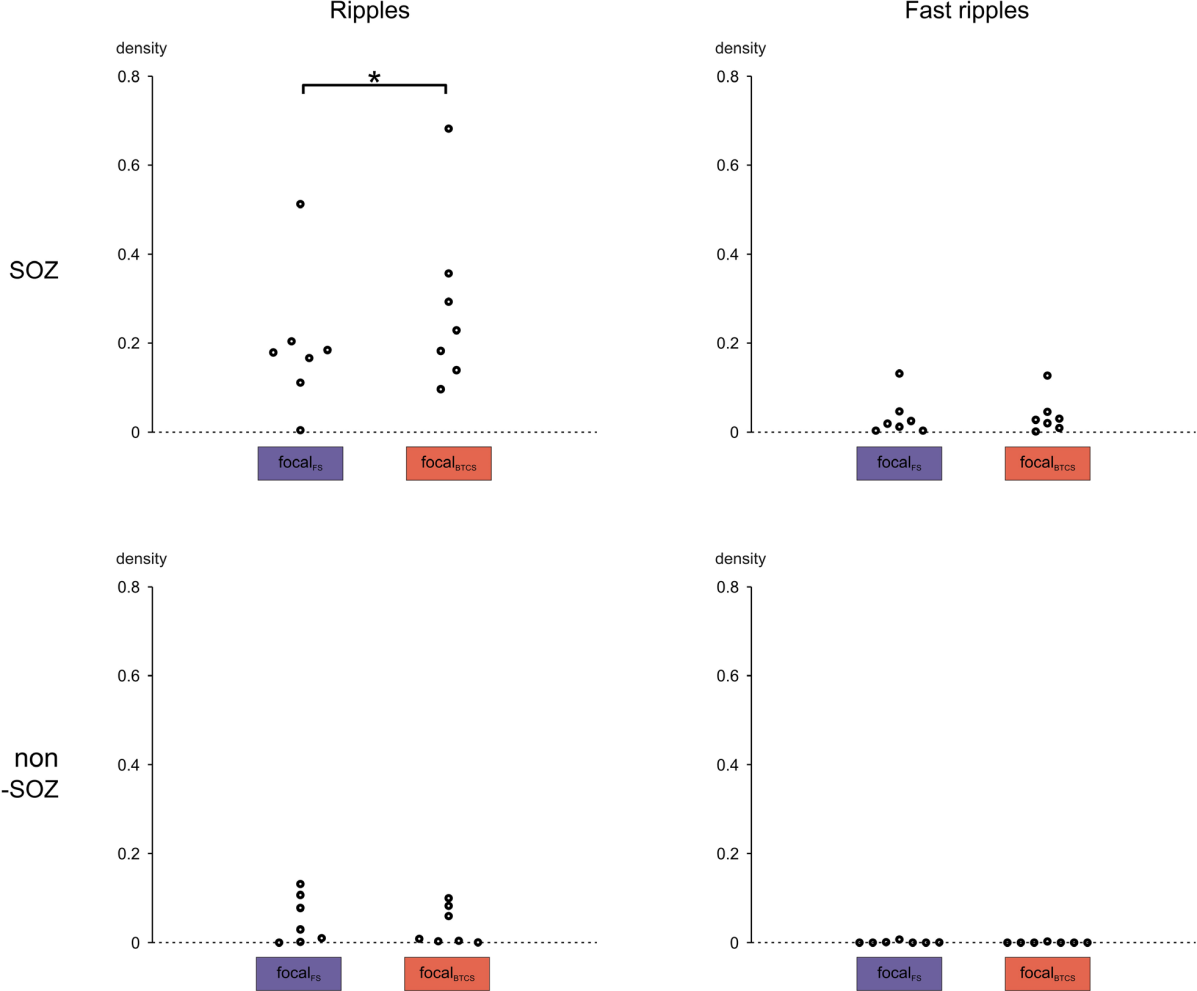
Predictive value of HFOs inside the SOZ regarding the progression to BTCS. Each dot corresponds to one patient. (Top, left) Prior to secondary onset of bilateral tonic‐clonic movements, ripple density was higher in BTCSs when compared to FSs. (Top, right) Fast ripple density was similar in the two groups. (Bottom, right) Ripple and (bottom, left) fast ripple densities were not significantly different. *indicates a statistically significant difference.

**Figure 6:**
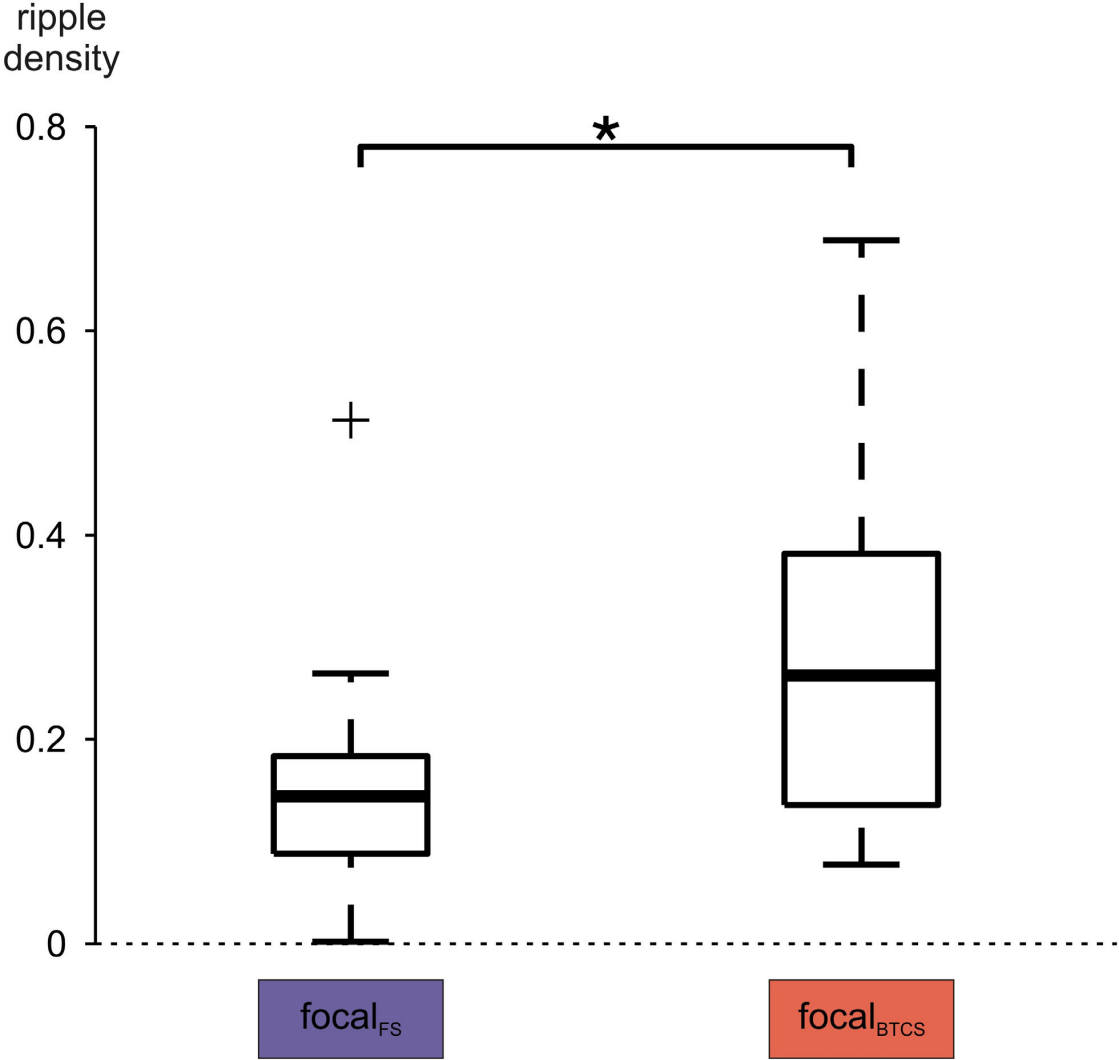
Prior to progression to a BTCS, SOZ ripple density was also higher in BTCSs if compared to FSs at the seizure level (*P* = 0.04). *indicates a statistically significant difference.

Finally, it was investigated whether FSs and BTCSs have different HFO subtype densities during comparable seizure stages. Ripple density inside the SOZ was higher in the focal part of BTCSs than in the focal part of FS. This difference was significant irrespective of whether groups were compared at the level of patients (*P* = 0.03, Wilcoxon matched‐pairs signed‐ranks test; Fig. 5) or seizures (*P* = 0.04; Wilcoxon rank sum test; Fig. 6). Fast ripple density, in contrast, was not significantly different (*P* = 0.69). In non‐SOZ channels, neither ripple (*P* = 0.30) nor fast ripple (*P* = 0.63) density differed significantly between FS and BTCS. The two seizure subgroups also had similar ripple and fast ripple densities in pre‐ and postictal intervals (Table 2). A characterizing feature of BTCS prior to actual onset of bilateral tonic‐clonic movements may thus be their higher ripple density inside the SOZ.

**Table 2:** Summary of comparison of HFO densities between FS and BTCS for pre‐ and postictal intervals. For clarity, only P values (Wilcoxon matched‐pairs signed‐ranks test) are specified.

	Ripples pre‐ictal	Ripples postictal	Fast ripples pre‐ictal	Fast ripples postictal
SOZ	0.94	0.13	0.69	0.84
Non‐SOZ	0.30	0.69	0.50	0.13

## Discussion

The main novel findings of this study are that (1) ripple and fast ripple densities increase inside the SOZ after seizure onset, and in remote areas after progression to a BTCS, (2) postictal SOZ ripple density drops below pre‐ictal levels and, most importantly, that (3) already prior to onset of bilateral tonic‐clonic movements, ripple density inside the SOZ is higher in BTCSs than in FSs.

### HFOs mirror onset and spread of seizure activity

We found that in human MTLE patients, ripple and fast ripple densities increase inside the SOZ following seizure onset. This finding is consistent with previous studies in a rodent model of MTLE^25^ and in a heterogeneous group of patients with a wide range of seizure onset patterns and underlying pathologies.^18^ The seizure onset‐associated increase remained confined to the SOZ – in remote areas,HFO subtype densities increased only after progression to a BTCS. Ripples and fast ripples are thus, in addition to their significance as interictal biomarkers,^11^, ^12^, ^14^, ^15^ also associated with ictal activity. Our study focused on frequencies traditionally defined for HFO analysis,therefore, and even though we took great care to exclude “false” HFOs,^17^ an effect on both HFO subtypes could theoretically have been due to wideband power increases. Alternatively, one might conclude that the cellular mechanisms underlying the two HFO subtypes, that is, synchronous firing and coordinating inhibitory currents (reflected by ripples)^3^, ^4^, ^5^ and out‐of‐phase firing of different pyramidal cell clusters (reflected by fast ripples),^7^, ^8^, ^9^ are also involved in the generation and spread of seizures.

### Postictal suppression of SOZ ripples

We report that ripple density inside the SOZ drops below pre‐ictal levels after the end of seizures. SOZ fast ripples and HFOs in remote regions were not significantly altered, suggesting that ripple‐generating networks are specifically silenced in the area that the seizure originated from. Interpretation of this finding is not trivial – even more – because it is still unclear which are the key mechanisms underlying seizure termination (see^26^ for a review). One possibility is that postictal hypoperfusion and hypoxia play a role,^27^ and that such metabolic stress is particularly critical for ripple‐generating parvalbumin‐positive basket cells due to their high energy demand.^28^ There is, though, also evidence for hyperperfusion after seizures^29^, ^30^, ^31^, thus, more complex neuronal interactions might as well be the cause. The suppression of ripples at least differentiates the postictal state from physiological slow‐wave sleep and might reflect that a patient with post‐seizure drowsiness is still in a pathological state associated with cognitive impairment – and not yet in a physiological sleep‐like mode. Whether our finding is also of lateralizing value for clinicians, as has been reported for postictal delta^32^ or EEG suppression,^33^ could be examined as part of a future investigation.

### Predictive value of SOZ ripples regarding progression to BTCSs

Our study reveals that SOZ ripple density in initial ictal intervals was higher if seizures progressed to BTCSs, than if they did not. No significant differences were found for fast ripples, or HFOs in remote areas. These results suggest that propagation is, at least to some degree, determined by network activity located inside the SOZ, rather than in remote areas. This would be consistent with previous data‐driven work underlining the impact of the SOZ on seizure spread.^16^ Whether progression to BTCSs is indeed promoted by HFOs or, as previous work choosing a different methodological approach suggests, primarily by slower activity^34^ may differ considerably between localization and type of a lesion. At the cellular level, there are at least two, somewhat divergent, interpretations of our finding: Prior to propagation of ictal activity, SOZ ripples could reflect rhythmic GABAergic currents that (1) support propagation of seizures, via synchronization of principal neurons, or (2) reflect compensatory inhibition. Irrespective of the underlying mechanisms, our results indicate that ripples might be a helpful biomarker for estimating the risk of progression to a BTCS. Taking into account the considerable overlap between FSs and BTCSs, however, one would not expect that an algorithm which solely relies on SOZ ripple density could be sufficiently performant for clinical application. We rather suggest that this parameter could be integrated in recently described multivariate approaches.^16^, ^34^

### Limitations and outlook

The current study is limited in some ways, and additional work is needed to fully investigate the role of HFOs during seizures. HFOs may reflect certain aspects of network activity, but for a complete picture of the underlying complex interactions, the occurrence of HFOs could be correlated to other frequency bands – at least to the neighboring ones, that is, high gamma^34^, ^35^ and very HFOs.^36^, ^37^ A combination with single‐unit analyses, as recently conducted for a subtype of seizure onsets in MTLE patients,^38^ might ultimately verify current hypotheses on cellular mechanisms.

The fact that we identified HFOs visually may limit applicability of this tool in a clinical routine setting. Sharp transients, wideband increases in signal power, and seizure‐associated artifacts still challenge automated detection, but with ongoing progress in the field of algorithm development, we might overcome this obstacle. If HFOs were to be used for prediction of BTCSs, solely relying on HFO density might not be promising. But our data suggest that this parameter could contribute to a device with acceptable performance, if combined, for example, with analyses of distinct morphological HFO features or applied on preselected seizure entities.^19^ Whether this becomes true, it can be concluded from the present study that HFOs mirror onset, termination, and also imminent spread of seizure activity, and that they can hence be considered as a biomarker reflecting not only epileptogenicity, but also different aspects of ictogenicity.

## Author Contributions

J. S. and J. J.: Conception and design of the study; J. S., N. B., D. L.‐P., M. D., A. S.‐B., J. J.: acquisition and analysis of data; J. S., N.B., and J. J.: drafting the figures and tables; J. S. and J. J.: wrote the manuscript.

## Conflict of Interest

None of the authors has any conflict of interest to disclose.
